# Anesthetic Management of Robot-Assisted Laparoscopic Surgery of Pheochromocytoma

**DOI:** 10.7759/cureus.53850

**Published:** 2024-02-08

**Authors:** Chhaya Suryawanshi, Sanya Varma, Subha Teresa J Vazhakalayil

**Affiliations:** 1 Anesthesiology, Dr. D. Y. Patil Medical College, Hospital & Research - Dr. D. Y. Patil Vidyapeeth University, Pune, IND

**Keywords:** robot-assisted surgery, robotic wrist, minimal invasive sugery, laparoscopy, pheochromocytoma

## Abstract

The latest trend shows a strong demand for minimally invasive surgery. The popularity of robot-assisted surgeries has increased because they eliminate many of the disadvantages of conventional laparoscopic methods.

However, compared to the conventional method of anesthesia treatment, robotic surgery may require adjustments in the way patients are positioned and the general arrangement of personnel and equipment.

Pheochromocytomas (PHEOs) are tumours of neural crest cells that make catecholamines. Early detection and appropriate treatment are required for PHEO. The best course of action for treating these tumours is an adrenalectomy. The last several years have seen a considerable advancement in surgical technology with the development of laparoscopic and robotic devices. By comprehending the fundamental elements of robotic surgical systems, anesthesiologists may better manage anesthesia and increase patient safety by being aware of these innovations.

## Introduction

A less common neuroendocrine tumor that produces catecholamines called pheochromocytoma (PHEO) may have an extra-adrenal or adrenal origin. Its manifestations include numerous symptoms, including sweating, headaches, palpitations, and hypertension [1]. PHEO is thought to occur in 0.05 to 0.1 percent of the general population. By testing for high levels of metanephrines in the body (blood or urine), PHEO is typically detected in the age group (40-50 years) with a slight female preference [2].

Minimally invasive surgical methods, mainly laparoscopic, have caused a revolution in surgery in recent years. Patients may experience smaller incisions during surgery, a decrease in pain, shorter hospital stays, and rapid recovery; however, the view is two-dimensional and uses giant tools with limited maneuverability [2].

In order to effectively treat problems during surgery and postoperatively, surgeons and anaesthesiologists must be aware of the precise pathophysiological changes that occur during surgery. Members of the interdisciplinary team involved in robotic surgery must also be more technologically organized, better and efficiently prepared, and can communicate clearly and thoroughly with each other. It is difficult to administer anesthesia due to the huge size of the tumor (2.2×2.0×2.9) and aggressive hemodynamic changes.

Anesthesia management in cases of pheochromocytoma undergoing robotic or other minimal access surgeries presents the clinician with a unique challenge due to sympathetic overactivity [3]. While the steps of securing arterial and central lines remain common across minimal access abdominal surgeries, the choice of induction agent as well as maintenance anesthesia varies. 

Catecholamines released during laryngoscopy and endotracheal intubation may aggravate PCC patients' hypertension and tachycardia. Extreme hypertension and tachycardia can cause arrhythmias and myocardial ischemia. Laryngoscopy and intubation elevate blood pressure and heart rate, although anesthesia lowers these [4].

Sevoflurane is the most commonly used inhalational drug for PCC resection anesthesia maintenance due to its low arrhythmogenic risk and favorable hemodynamic profile.

Similarly, peritoneal insufflation and tumor manipulation during laparoscopic excision can rapidly release catecholamine, causing hypertension and tachycardia. Hypertension crises can cause myocardial ischemia or stroke [5]. Nitroprusside, nitroglycerine, and calcium channel blockers like nifedipine and clevidipine treat hypertension and help manage coronary artery spasms secondary to excess release of catecholamines in PCC.

For PCC patients, beta-blockers should be considered after α-blocker medication. Catecholamine-induced α-agonism can lead to acute hypertension, increased afterload, and myocardial oxygen demand if unopposed. It is also important for the anesthetist to avoid labetalol as it may cause unopposed α-receptor agonism from circulating catecholamines, leading to hypertensive crises due to its β-adrenergic antagonism [6].

Variable anesthesia depth optimizes hemodynamic responsiveness. Tumour blood vessel ligation may sharply lower blood pressure. Endogenous catecholamine levels drop rapidly in PCC patients due to chronic alpha-adrenergic receptor downregulation, which can cause refractory hypotension, obviating the need for vasopressors. 

There are very few studies that have assessed the above factors or parameters in robotic adrenalectomy. Hence, through this case, we would like to highlight our experience and provide a brief literature review. 

## Case presentation

A 72-year-old female who weighed 75 kg presented with a hazy vision, an excruciating headache, and pain in the abdomen. Preoperative testing showed an enlarged abdomen, preoperative blood pressure of 140/90 mmHg, and 100% SpO_2_._​​​_

Upon abdominal examination, a firm-feeling bulge that started on the left side of the abdomen and crossed the midline was visible. The results of the testing revealed a normal hemoglobin level of 9 gm%. A focal heterogeneously growing lesion in the left adrenal gland that appeared to have a malignant etiology was seen on computed tomography. A small F-18 fluorodeoxyglucose well-defined soft tissue density lesion involving the left adrenal s/o pheochromocytoma was visible on a whole-body positron emission tomography (PET) scan.

**Figure 1:**
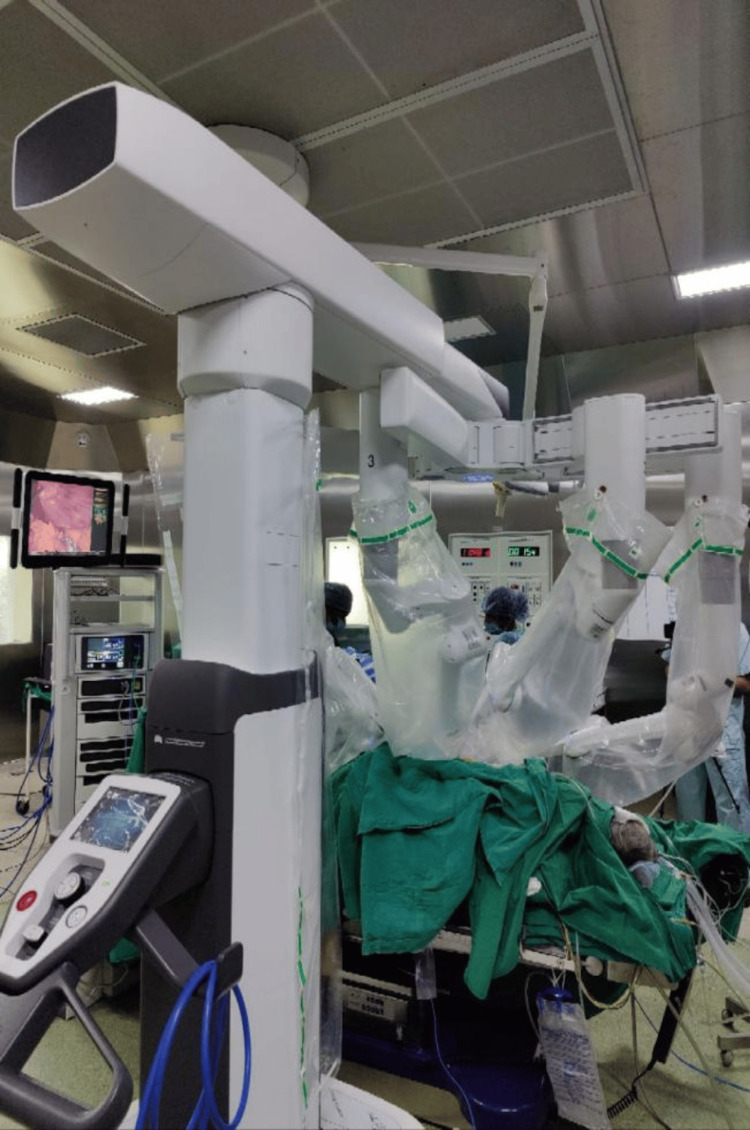
Fourth Generation Da Vinci Robotic System

**Figure 2:**
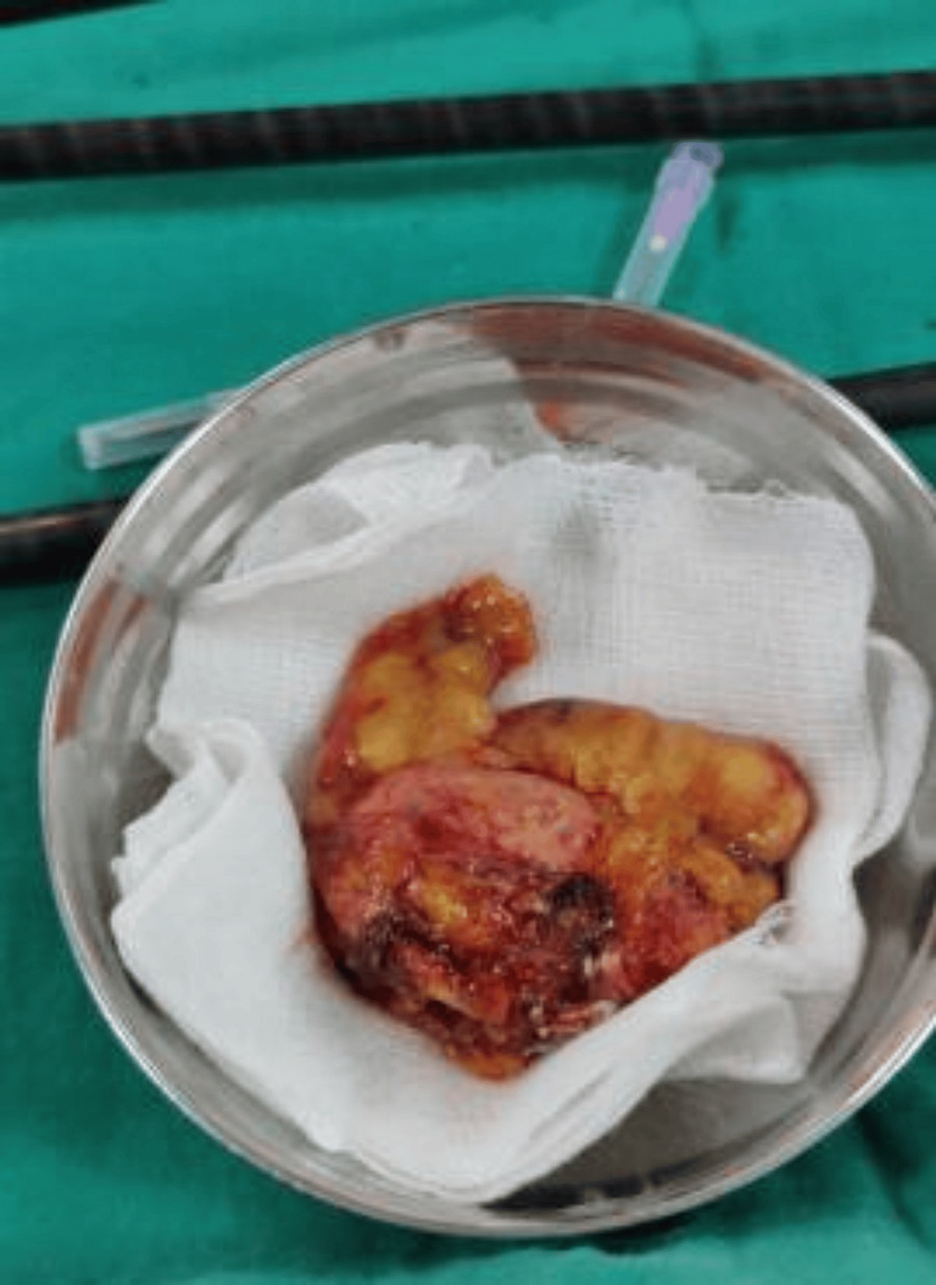
Pheochromocytoma tumor size (2.2×2.0×2.9)

**Figure 3:**
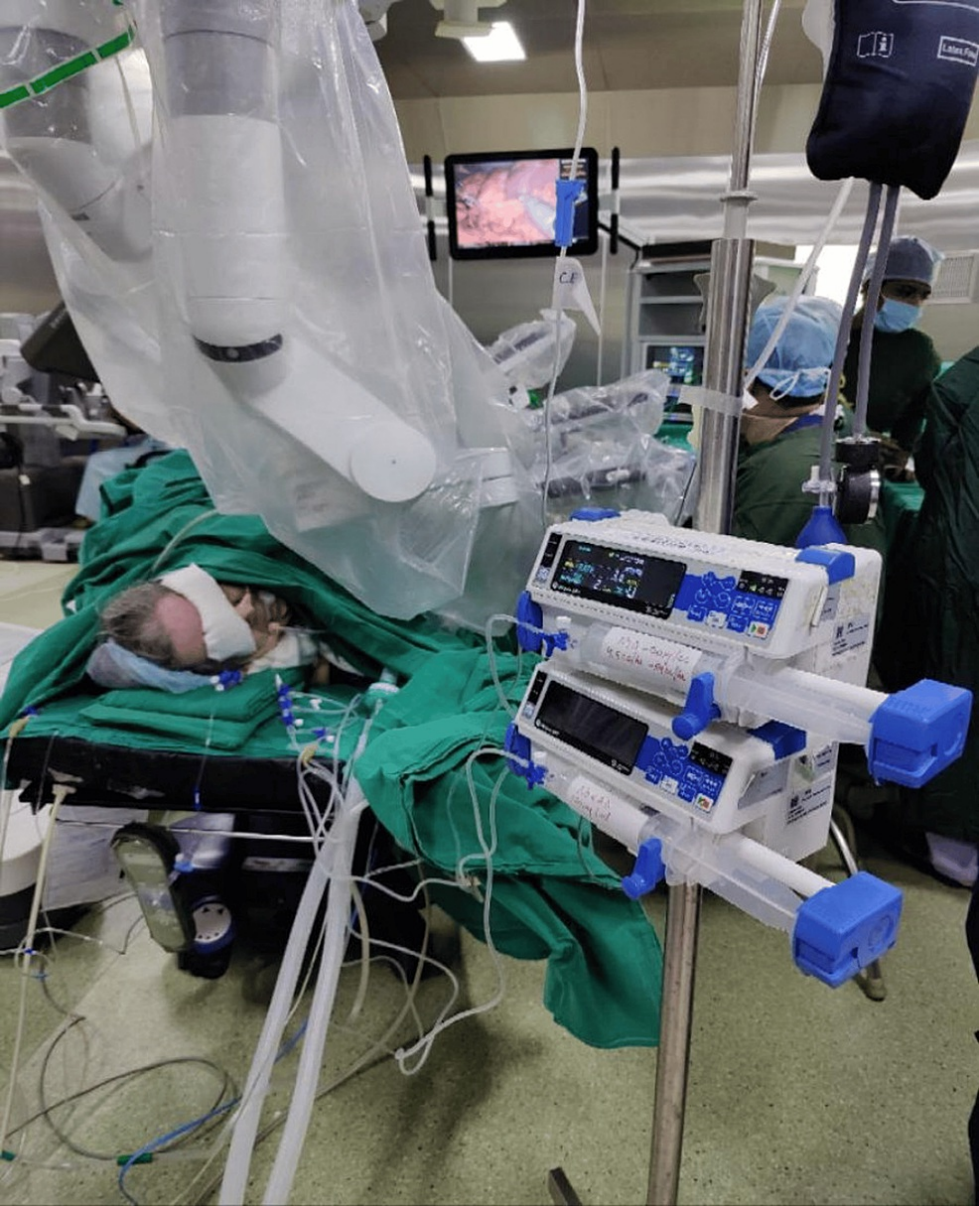
Intraoperative robot-assisted laparoscopic surgery of pheochromocytoma

The patient was taking tablet phenoxybenzamine 10 mg twice daily before the surgery as a means to control intraoperative blood pressure fluctuations. On the day of the operation, blood and blood products were ordered, standard American Society of Anesthesiologists (ASA) monitors with train of four (TOF) and bispectral index (BIS) monitoring were attached, and high-risk/ICU consent was acquired. Fentanyl 100mcg was injected after the patient had been premedicated with IV glycopyrrolate (0.004 mg/kg) and IV midazolam (0.02 mg/kg). She was preoxygenated with 100% O^2^. Propofol (2 mg/kg) and IV vecuronium (0.1 mg/kg) were administered, and the patient was intubated with a size 7.5 cuffed endotracheal tube. The tube was fixed after confirming bilateral and equal airway entry by auscultation. A triple-lumen 7 French central catheter was positioned in the right internal jugular vein (IJV), and an 18G epidural catheter was inserted at the level L1-L2 in the left lateral position for postoperative analgesia. The arterial line was secured in the right radial artery. The patient was kept on O_2_:AIR (50:50) with 0.8 isoflurane and a minimum alveolar concentration (MAC) of 1.2. To maintain blood pressure and perioperative analgesia, ropivacaine 0.2% was started. Blood pressure was kept constant during surgery at 90-100/60-70 mmHg, and a mean arterial pressure (MAP) of 60-65mmHg was maintained. An adrenalectomy (Figure 1, 2, 3) was done once the tumor had been seen. There was blood loss of 50 ml and urine output of 200 ml. Total IV fluids of 1000 ml, injections of 1 mg of dexamethasone, and 15 mg/kg of paracetamol was given. A 0.5 mcg/kg/min infusion of nitroglycerin (NTG)and a 0.05 mcg/kg/min infusion of noradrenaline (NORAD) were started to keep the blood pressure stable.

Following surgery, the patient received a reversal injection of 0.4 mg glycopyrrolate and 2.5 mg neostigmine to reverse the effects of muscle relaxant vecuronium. Extubating the patient went smoothly and without any incident. For observation, the patient was taken to the critical care unit.

## Discussion

Chromaffin cells of the adrenal medulla give rise to an unusual catecholamine carcinoma known as malignant PHEO [2]. Surgical treatment of malignant PHEO is an adrenectomy or debulking procedure [3]. Open surgery, laparoscopic surgery, and robotic surgery are all options. In their 2015 study, Goffredo et al. examined trends in the use of minimally invasive surgery (MIS) for malignant PHEOS in the United States and compared the short-term effects of Minimally invasive surgery with open adrenalectomy [4]. Here, we elaborate on the techniques and challenges we faced as anesthetists in this particular case.

Alpha-1 receptor blocking is used frequently before surgery in order to minimize the occurrence of an increase in blood pressure intraoperatively due to increased amounts of catecholamines in the bloodstream. The development of pneumoperitoneum, adrenal manipulation, and endotracheal intubation are all three perioperative phases that are at high risk for hemodynamic instability [5,6]. To reduce intraoperative hemodynamic instability, alpha adrenergic inhibition has been widely recognized as a management technique before surgery [7]. The same was employed in our study as well, and throughout the conduct of the surgery, we faced minimal hemodynamic variation. More recently, other preoperative medical preparation regimens using calcium channel blockers or other drugs have also been proposed [5].

For our patient, the standard robotic system and console Fourth Generation Da Vinci Robotic System (Intuitive Surgical Inc., Sunnyvale, California) was utilized. It is essential to know that despite the advantages, robotic surgery is not without its fair share of problems. Pertaining to the specific docking process it involves positioning of the patient and insertion of the ports before attaching the instrument arms of the patient cart to the patient. After docking, even little alterations to the operating table's position might be extremely dangerous for the patient. Any anesthetic technique or new vascular access insertion will be extremely challenging [8]. Therefore, as a protocol in our institute, the anesthesia workstation has a vantage point of the monitors and easy access to the drugs.

Planning and adjusting the administration of anesthesia should be done in accordance with the patient's state of health. As part of the standard introduction to general anesthesia involves securing the airway by using the right-sized endotracheal tube (ET) tube, which should be properly inserted, and the position of the tube should be checked by auscultation, after which it should be properly taped and fixed, a high probability of the tube migrating into the unventilated lung and collapsing, causing hypoxia, is a possibility. A three-point ET cuff palpation approach is easy to use, reliable, and successful in decreasing the chances of endobronchial tube movement during robotic pelvic surgery, according to a recently published study [9]. ET tube position should also be checked regularly. Tidal volume should be set between 6 and 8 ml/kg, and Pplat should not be greater than 30 cm H_2_O. Before that, ventilation with lung protection should be used. 

## Conclusions

With superior control and greater dexterity, robotic surgery has significantly changed how surgeons perform surgery. The advantages of robotic surgery over laparoscopy in adrenalectomy have been extensively studied. Robotic surgery is becoming more common and is being utilized more frequently in a variety of disciplines. Anesthesiologists must therefore stay up to date on new information and be ready to offer these patients superior anesthesia care.
